# Application of Salicylic Acid Improves the Production of Medicinal Components in *Mucuna macrocarpa* Wall by Regulating Endogenous Hormone and Nutrient Balance

**DOI:** 10.3390/plants14071023

**Published:** 2025-03-25

**Authors:** Yulan Hua, Xianyuan Pan, Li Tian, Yuanyuan Xu, Mei Yang, Rongyan Deng

**Affiliations:** 1 Guangxi Key Laboratory of Forest Ecology and Conservation, Guangxi Colleges and Universities Key Laboratory for Cultivation and Utilization of Subtropical Forest Plantation, College of Forestry, Guangxi University, Nanning 530004, China; 2209302004@st.gxu.edu.cn (Y.H.); 2209392047@st.gxu.edu.cn (L.T.); yuanyuanxu@gxu.edu.cn (Y.X.); 2 Guangxi Zhuang Autonomous Region State-Owned Qipo Forest Farm, Nanning 530225, China; pxianyuan2018@163.com

**Keywords:** *Mucuna macrocarpa* wall, salicylic acid, total flavonoids, total phenolics, endogenous hormone, nutrient balance

## Abstract

The *Mucuna macrocarpa* Wall, a traditional Chinese medicinal plant, exhibits significant cultivation-dependent variations in the accumulation and yield of its medicinal components. Salicylic acid (SA) has demonstrated the potential to regulate plant growth, which can be strategically used to enhance medicinal yield, offering a promising approach for high-yield cultivation in medicinal plants. This study aimed to investigate the changes in the medicinal components of *Mucuna macrocarpa* seedlings (4 months old) at different concentrations of SA (0, 0.1, 0.5, 0.9, and 1.3 mM) in a pot experiment. The results indicate that SA significantly increased the basal diameter (BD) by 2.9% to 20.61% and the total biomass (TB) by 14.28% to 48.57%. Notably, SA treatments resulted in alterations in the endogenous hormone content, including indole-3-acetic acid (IAA), abscisic acid (ABA), gibberellin A_3_ (GA_3_), and trans-zeatin-riboside (ZR), and the balance in leaves. SA regulated the content and balance of nitrogen (N), phosphorus (P), and potassium (K) in all organs, and K content and K: P in roots, stems, and leaves was significantly higher than that of the control under 0.9 mM SA treatment. Crucially, SA significantly enhanced the content of bioactive compounds. The total phenolic content (TPC) and total flavonoid content (TFC) in stems peaked at 0.9 mM (14.89 mg·g^−1^ and 3.73 mg·g^−1^, respectively), which were 11.87% and 11.68% higher than those in the control. Moreover, compared to the control, SA treatments increased total phenolic production by 20.00% to 61.45% and total flavonoid production by 3.89% to 90.56%. In addition, 0.9 mM SA was found to be more effective than other treatments for increasing total phenolic and d total flavonoid content. In summary, this study investigated the effect of SA as an eco-friendly elicitor to improve the total phenolic and total flavonoid production of *Mucuna macrocarpa*.

## 1. Introduction

The *Mucuna macrocarpa* Wall, a precious traditional medicinal plant of the Fabaceae family [1], is naturally distributed in Guangdong, Guangxi, Yunnan, and Guizhou, China. With the rising demand for *Mucuna macrocarpa* (*M. macrocarpa*) and growing resource scarcity, enhancing its quality and yield through environmentally friendly methods has become a critical challenge. To date, the studies on *M. macrocarpa* have predominantly focused on its medicinal components [2,3] and pharmacological properties [4], while strategies to enhance growth, medicinal quality, and yield of *M. macrocarpa* remain unexplored. The seedling stage represents the initial phase of the plant life cycle. Vigorous seedlings exhibit accelerated growth due to enhanced nutrient uptake and energy accumulation, thereby establishing a critical foundation for optimizing final yield and phytochemical quality. Consequently, promoting seedling growth through targeted cultivation is not only a prerequisite for high-yield cultivation of *M. macrocarpa* but also essential for early-stage yield exploitation.

Phytohormones are key small molecules that regulate plant growth, physiology, secondary metabolism, and stress responses. The application of exogenous hormones can change the hormone levels in plants, modulate the physiological metabolism of various plant parts, and direct plant growth and development [5]. Studies have shown that the application of exogenous hormones effectively increased the yield of crops such as *Zea mays* L. [6], *Oryza sativa* L. [7], and others. Furthermore, plant hormones can promote plant growth and are essential for achieving optimal plant development. Salicylic acid (SA) is a phytohormone [8] that is involved in various biochemical and physiological activities during plant growth [9,10], such as regulating plant response to environmental stress [11]. Previous studies have demonstrated that SA can enhance photosynthesis in plants by modulating the synthesis and accumulation of photosynthetic pigments [12,13]. Moreover, SA has been observed to enhance root vigor [14], stimulate root growth, and increase plant yield [15]. Similarly, Pirasteh-Anosheh [16] also showed that exogenous application of SA not only increased the grain yield of barley and wheat but also improved their salt tolerance. In a study on wheat, Colak [17] observed that SA boosted photosynthetic pigment levels and promoted root and leaf growth.

Additionally, SA is involved in the regulation of the synthesis of plant secondary metabolites and affects the accumulation of total flavonoids, total phenols, and other active substances [18,19]. Dai [20] found that SA induced the synthesis of citrus flavonoids. Previous studies have shown that SA increases the total flavonoid and total phenolic content in the adventitious roots of *Cannabis sativa* L. [21]. Although there are numerous studies on the application of SA in plant cultivation, the synergistic effects of SA on endogenous hormone balance, multi-organ nutrient allocation, and medicinal compound accumulation in medicinal plants remain poorly explored. Specifically, studies on SA-driven enhancement in medicinal productivity in *M. macrocarpa* are scarce.

In this study, four-month-old *M. macrocarpa* seedlings were treated with gradient concentrations of SA to investigate its effects on seedling growth, endogenous hormone levels, organ-specific nutrient distribution, and medicinal compound production, with a focus on elucidating the interrelationships among endogenous hormones, nutrient homeostasis, and the principal bioactive constituent accumulation. This research will provide a theoretical basis and a specific SA application protocol for the high-yield cultivation of *M. macrocarpa* as well as other medicinal plants.

## 2. Results

### 2.1. Effect of SA Concentrations on the Growth and Biomass of M. macrocarpa

**Figure 1:**
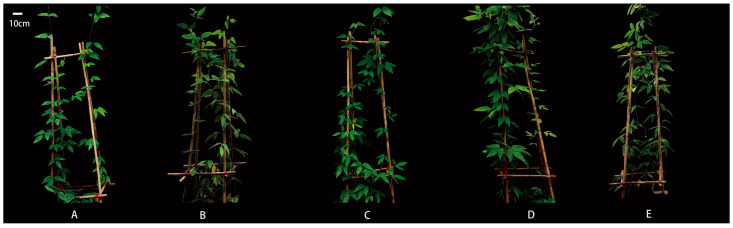
Images of *M. macrocarpa* under different SA concentration (mM) treatments. (**A**–**E**) represent 0, 0.1, 0.5, 0.9, and 1.3 mM, respectively.

SA treatments considerably increased the growth of *M. macrocarpa*, as confirmed by the basal diameter and biomass (Table 1). At 0.9 mM SA concentration, the basal diameter reached a maximum of 8.31 mm, which was 20.61% higher than that of the control (0 mM) (*p* < 0.01); however, the basal diameter started to decrease at 1.3 mM SA concentration. When compared to the control, SA also enhanced the biomass of *M. macrocarpa*. Root biomass and total biomass reached their maximum values (25.36 g and 78.64 g, respectively) at an SA concentration of 1.3 Mm, and were 55.48% and 48.57% higher than the control (*p* < 0.01), respectively. Both stem biomass and leaf biomass peaked at 0.9 mM SA treatment (27.97 g and 26.19 g, respectively), representing increases of 44.25% and 52.09% over the control (*p* < 0.01). The images of *M. macrocarpa* treated with SA (0, 0.1, 0.5, 0.9, and 1.3 mM) are presented in Figure 1A–E.

### 2.2. Effect of SA Concentrations on Endogenous Hormone Content in Leaves of M. macrocarpa

#### 2.2.1. Content of IAA, ABA, GA_3_, and ZR

The content of IAA, GA3, ZR, and ABA in leaves of *M. macrocarpa* changed significantly under SA treatments (Table 2). Both IAA and ABA reached their maximum values at 0.5 mM SA (57.08 ng·g^−1^ and 160.14 ng·g^−1^, respectively). At this SA concentration, IAA content was 23.79% higher (*p* < 0.01) and ABA was 2.29% higher (*p* < 0.05) than those in the control. In comparison to 0 mM, SA raised the levels of ZR and GA_3_. The GA_3_ content was 1.24–26.03% higher than that of the control, and ZR content was 4.19–52.93% higher than that of the control.

#### 2.2.2. Ratio of Endogenous Hormones

Compared with the control (0 mM SA), endogenous hormone ratios were significantly altered under SA treatments (Table 3). The ratios of IAA:ABA, GA_3_:ABA, ZR:ABA, and (IAA + GA_3_ + ZR):ABA reached the highest level at 0.1 mM SA concentration (0.4080, 0.0484, 0.0712, and 0.5275, respectively), representing increases of 38.47%, 56.63%, 86.39%, and 44.96% compared to the control (*p* < 0.01). However, these ratios gradually declined as SA concentration increased; in particular, the IAA:ABA ratio at 0.9 mM was even lower than that of the control.

### 2.3. Effect of SA Concentrations on Nutrient Content and on the Stoichiometric Ratios of M. macrocarpa

#### 2.3.1. N, P, and K Content

**Figure 2:**
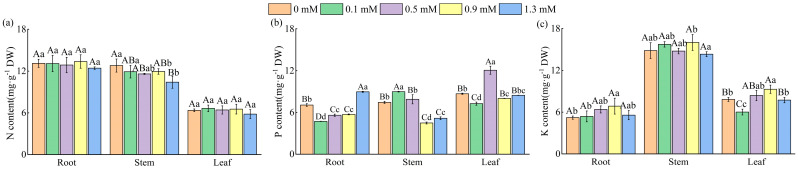
N (**a**), P (**b**), and K (**c**) nutrient content in in root, stem, and leaf. Data are expressed as mean ± standard deviation of three biological replicates. Different capital letters for the same organ in the figure indicate highly significant differences between treatments (*p* < 0.01), and different lowercase letters indicate significant differences between treatments (*p* < 0.05).

The nitrogen (N), phosphorus (P), and potassium (K) content of different organs exhibited different response patterns to SA (Figure 2a–c). Compared to the control, stem N content decreased by 6.67% to 18.67% under SA treatments (Figure 2a), whereas no significant differences in N content were observed in roots or leaves across treatments (*p* > 0.05).

The P content in different organs showed varying responses to SA (Figure 2b). Root P content significantly decreased by 33.24%, 20.74%, and 19.05% under 0.1, 0.5, and 0.9 mM SA treatments, respectively, compared to the control; however, it increased by 26.83% at 1.3 mM SA (*p* < 0.01). In stems, low SA concentrations (0.1 and 0.5 mM) increased P content by 20.62% and 6.00% (*p* < 0.05), whereas higher concentrations (0.9 and 1.3 mM) caused significant reductions of 39.76% and 30.55% (*p* < 0.01). Leaf P content peaked at 0.5 mM SA (12.07 mg·g^−1^), demonstrating a significant increase of 38.93% compared to the control.

The potassium (K) contents in roots, stems, and leaves all peaked at 0.9 mM SA, with increases of 31.80%, 7.95%, and 18.62%, respectively, compared to the control (*p* < 0.05) (Figure 2c).

#### 2.3.2. Ratio of N:P, N:K, and K:P

**Figure 3:**
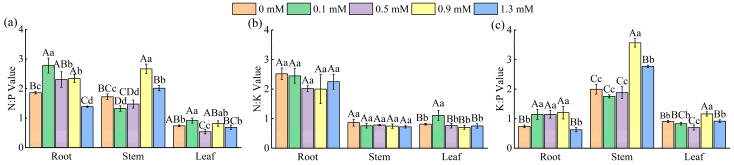
Characteristics of the N:P (**a**), N:K (**b**), and K:P (**c**) ratio changes in root, stem, and leaf. Data are expressed as mean ± standard deviation of three biological replicates. Different capital letters for the same organ in the figure indicate highly significant differences between treatments (*p* < 0.01), and different lowercase letters indicate significant differences between treatments (*p* < 0.05).

As shown in Figure 3a, the N:P ratios of roots, stems, and leaves peaked at 0.1 mM, 0.9 mM, and 0.1 mM, respectively (2.78, 2.67, and 0.91), corresponding to increases of 49.46%, 54.65%, and 9.50% compared to the control (0 mM SA) (*p* < 0.05).

The N:K ratios of different organs of *M. macrocarpa* exhibited different trends with increasing SA concentrations (Figure 3b). Root and stem N:K ratios showed no significant differences (*p* > 0.05) across SA treatments, whereas leaf N:K peaked at 1.11 at 0.1 mM SA, demonstrating a significant increase (*p* < 0.01) compared to other concentrations.

The K:P ratios of different organs exhibited the order: stems > roots > leaves (Figure 3c). Root K:P reached a maximum value (1.20) at 0.9 mM SA, which was 62.16% higher than the control (*p* < 0.01). The K:P values of stems and leaves also peaked at 0.9 mM (3.57 and 1.16, respectively), with increases of 79.4% and 28.89% relative to the control (*p* < 0.01).

### 2.4. Effect of SA Concentrations on the Medicinal Components of M. macrocarpa

#### 2.4.1. Total Phenolic Content (TPC) and Total Flavonoid Content (TFC) in Various Organs

**Figure 4:**
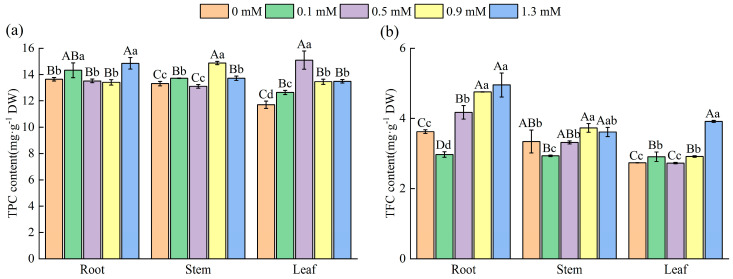
TPC (**a**) and TFC (**b**) content in root, stem, and leaf. Data are expressed as mean ± standard deviation of three biological replicates. Different capital letters for the same organ in the figure indicate highly significant differences between treatments (*p* < 0.01), and different lowercase letters indicate significant differences between treatments (*p* < 0.05).

The TPC exhibited organ-specific divergence under SA treatments (Figure 4a). The TPC of roots reached its maximum value at 1.3 mM SA, with an 8.86% increase compared to 0 mM SA (*p* < 0.05). The TPC of stems and leaves reached the maximum values (14.89 mg·g^−1^ and 15.10 mg·g^−1^, respectively) at 0.9 mM and 0.5 mM, respectively, both significantly higher than those at 0 mM (*p* < 0.05).

SA (0.9 and 1.3 mM) significantly increased TFC in all three organs compared to the control (Figure 4b). The TFC of roots and leaves peaked at 1.3 mM (4.95 mg·g^−1^ and 3.91 mg·g^−1^, respectively), with increases of 36.74% and 43.07% compared to those at 0 mM (*p* < 0.01). Under the 0.9 mM SA treatment, the TFC of the stems reached the maximum value (3.73 mg·g^−1^), which was 11.68% higher than 0 mM (*p* < 0.01), indicating that exogenous SA differentially modulated TPC and TFC across organs of *M. macrocarpa*.

#### 2.4.2. Total Phenolic Production (TPP) and Total Flavonoid Production (TFP) of the Whole Plant

**Figure 5:**
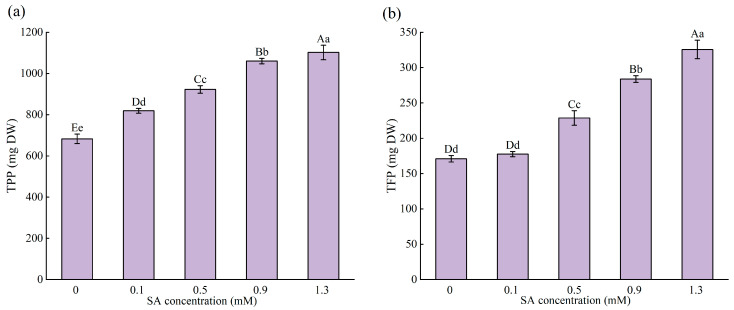
Changes in TPP (**a**) and TFP (**b**) under different SA concentration treatments. Data are expressed as mean ± standard deviation of three biological replicates. Different capital letters for the same organ in the figure indicate highly significant differences between treatments (*p* < 0.01), and different lowercase letters indicate significant differences between treatments (*p* < 0.05).

The TPC and TFC of the different organs and the biomass of each organ were used to calculate the total phenolic and total flavonoid yields of the whole plant and to analyze the changes in TPP and TFP at different concentrations of SA (Figure 5a,b). It was found that SA significantly increased TFP and TPP. Compared with the control, SA increased TPP by 20.00% to 61.45% and TFP by 3.89% to 90.56%. Although the highest levels of TPP and TFP were observed at 1.3 mM, the increments of TPP between two neighboring treatments began to decelerate at this treatment.

### 2.5. Correlation Analysis

To investigate the relationships among plant growth, endogenous hormones, plant nutrient elements in various organs, total phenolics, and total flavonoids, Pearson correlation analysis was performed (Table 4). Pearson correlation analysis indicated that (*p* < 0.05) the growth indicators (basal diameter, BD; total biomass, TB) were strongly correlated with stem total phenolic content (STPC), stem total flavonoid content (STFC), total phenolic production (TPP), and total flavonoid production (TFP). The endogenous plant hormone ABA showed a positive correlation with root total flavonoid content (RTFC) and STFC (*p* < 0.05). IAA was significantly negatively correlated with LTPC and STFC (*p* < 0.05) and strongly positively correlated with STPC (*p* < 0.01). Stem N content (SN) was negatively correlated with root total phenolic content (RTPC), TPP, and TFP (*p* < 0.05), and showed a significant negative correlation with LTFC (*p* < 0.01). Stem P content (SP) was strongly negatively correlated with STPC, RTFC, STFC, TPP, and TFP (*p* < 0.01). Stem K content (SK) was significantly positively correlated with STPC (*p* < 0.05), whereas leaf K content (LK) was very strongly positively correlated with RTFC and STFC (*p* < 0.01).

### 2.6. Membership Function Analysis

In order to further investigate the effects of different concentrations of SA on the yield of the total phenolics and total flavonoids, the treatments were subjected to a membership function analysis based on the correlation of each indicator with TPP and TFP (Table 5) to identify the treatment that is most conducive to achieving the maximized production of *M. macrocarpa*. As shown in the table, the mean membership function values of the five treatments (0, 0.1, 0.5, 0.9, and 1.3 mM) were 0.276, 0.375, 0.614, 0.667, and 0.506, respectively. All SA-treated groups exhibited higher membership values than the control (0 mM SA), with the comprehensive ranking as follows: 0.9 mM > 0.5 mM > 1.3 mM > 0.1 mM > 0 mM.

## 3. Discussion

Previous studies have shown that SA is widely distributed in plants and alters physiological and metabolic responses, thereby affecting plant growth and development [22]. In this study, we observed that SA markedly increased basal diameter, stem biomass and leaf biomass at 0.9 mM, but these parameters started to decrease at 1.3 mM SA. These data suggest that excessive SA concentrations exerted an inhibitory effect on plant growth, thus slowing down the growth of leaves and stems, as reported in a previous study in *Capsicum annuum* [23]. We also found that SA treatments increased the levels of TPP and TFP in *M. macrocarpa*, with a significant positive correlation between these levels and the growth indicators of basal diameter and total biomass. These findings further suggest that SA could provide energy and space for the synthesis of phenols and flavonoids in plants by promoting plant growth and biomass accumulation [24]. However, 1.3 mM SA had a negative impact on the content of total phenolics and total flavonoids in stems. Therefore, it can be inferred that excessive concentrations of SA induced hormonal stress, which inhibits growth rate and biomass accumulation, consequently affecting metabolic processes [25].

The synthesis of phenols and flavonoids is influenced by many factors. For example, studies have shown that SA can affect the interactions of endogenous hormones and the nutrient balance within the plants, and ultimately influence the total phenolic and total flavonoid content and accumulation [26]. Endogenous hormones exhibit distinct regulatory functions in plant growth modulation and developmental processes. IAA, GA_3_, and ZR are key growth-promoting hormones [27,28], whereas ABA inhibits plant cell growth, induces seed dormancy and leaf abscission, participates in stress resistance enzyme synthesis [29], and regulates key genes associated with flavonoid biosynthesis [30].

In this study, SA significantly increased these hormone ratios compared to the control. However, all ratios decreased with rising SA concentrations, indicating that low SA concentrations favor the synthesis of growth-promoting endogenous hormones, while excessive SA concentrations trigger physiological stress acclimation, stimulating ABA biosynthesis through phytohormone crosstalk. Notably, ABA peaked at 0.5 mM, whereas IAA, GA_3_, and ZR reached their maximum values at 0.5, 0.1, and 0.9 mM, respectively, and then began to decline, which was similar to the results of the endogenous hormones reported for *Blumea balsamifera* [31] in response to SA. These findings suggested that 0.5 mM SA increased ABA accumulation, subsequently leading to a decrease in the levels of the endogenous hormones IAA, GA_3_, and ZR [32]. ABA can regulate flavonoid synthesis-related gene expression via shared signaling pathways or transcription factors, thereby influencing both the biosynthetic pathways of phenolic compounds and the total content of phenolics and flavonoids [33]. In this study, ABA exhibited a positive correlation with the total flavonoid content of roots and stems, suggesting that SA-promoted ABA synthesis in *M. macrocarpa* synergistically modulated flavonoid biosynthesis, ultimately elevating flavonoid levels. This hypothesis is corroborated by findings from previous investigations on *Oryza sativa* [34], *Scutellaria baicalensis* [35], and *Ipomoea batatas* [36].

N, P, and K are the three major nutrients necessary for plant growth and are the critical factors for plant nutrient absorption, cycling and utilization, and qualitative development [37]. In this study, SA exhibited organ-specific effects on N, P, and K distribution: the 0.1 mM treatment enhanced stem P content, whereas 0.9 mM SA promoted root N accumulation and increased K content in roots, stems, and leaves. These patterns align with previous studies on *Brassica juncea* [38] and *Vallisneria natans* [39]. Correlation analysis revealed a negative correlation between stem P and the production of total phenolics and total flavonoids, suggesting an existing enzyme activity regulation mechanism between P and the accumulation of total phenolics and total flavonoids. This may also be due to the decrease in the IAA:ABA ratio at medium to high SA concentrations, affecting the expression of P transporter proteins, which may put the plant into a low-phosphorus state and lead to the production of more reactive oxygen species (ROS) [40]. A comparable mechanism was reported in *Artemisia argy* under low- and high-P treatments, where antioxidant production (e.g., total phenolics and total flavonoids) increased [41].

Plant growth status can be assessed through N:P, N:K, and K:P ratios. For instance, it is widely accepted that plants are predominantly nitrogen-limited when N:P < 14 [42], whereas phosphorus limitation dominates at N:P > 16 [43]. Additionally, potassium limitation typically occurs when N:K > 2.1 and K:P < 3.4 [44]. In this study, N:P ratios of all *M. macrocarpa* organs were less than 14, indicating N-limitation. Root N:K > 2.1 and K:P < 3.4 further suggested K-limitation in roots. SA application initially increased root N:P and K:P ratios, but these values declined at higher concentrations (1.3 mM SA) to even less than those of the control. These results demonstrate that SA modulated nutrient balance in *M. macrocarpa*, alleviating N-limitation at lower concentrations but exacerbating N- and K-limitation at excessive doses. This can be attributed to the ability of SA to reduce Na^+^ uptake and enhance K^+^ influx via H ^+^-ATPase activation [45], while promoting N assimilation and utilization through nitrate reductase upregulation [46], thereby altering the nutrient homeostasis. In contrast, excessive concentrations of SA inhibited the activity of enzymes related to N and K synthesis. Notably, N is a key determinant of total phenolic and total flavonoid content [47,48]. Under low nitrogen conditions, plants increased antioxidant production (e.g., total phenolics and total flavonoids) to counteract oxidative stress [49]. In this study, negative correlations between N and the production of total phenolics and total flavonoids under nutrient-limited conditions suggested that SA enhanced antioxidant systems in *M. macrocarpa*, thereby promoting secondary metabolite accumulation.

In the context of seedling cultivation, applying exogenous SA at appropriate concentrations enhanced the growth rate of *M. macrocarpa*. Based on a comprehensive membership function analysis, the optimal concentration for high-yield cultivation was determined to be 0.9 mM. This study provides preliminary yet significant insights into *M. macrocarpa* cultivation. Building on these findings, future studies should focus on elucidating the molecular mechanisms governing the biosynthesis of medicinal compounds in this species.

## 4. Materials and Methods

### 4.1. Plant Material and Growth Conditions

Four-month-old *M. macrocarpa* seedlings were provided by the Guangxi State-owned Qipo Forest Farm, and pot experiments were carried out in the suburbs of Nanning City, Guangxi Zhuang Autonomous Region, China (108°12′25″ E, 22°42′18″ N). It belongs to the subtropical monsoon climate zone, with high temperatures ranging from 16 to 31 °C and low temperatures from 7 to 22 °C, with an average annual rainfall of 1304.2 mm. During the cultivation of seedlings, the environmental conditions in the greenhouse were 72% relative humidity, 20.8 °C, and a 12 h photoperiod.

In this study, the test seedlings were cultivated in plastic pots (base diameter 170 mm, height 200 mm), and a 1.7 m stand was placed adjacent to each pot to provide a climbing structure. The cultivation experiment utilized a mixed substrate composed of yellow sub-soil, peat soil, and cocopeat in a volumetric ratio of 5:3:2. The organic carbon, total nitrogen, total phosphorus, and total potassium contents of the mixed substrate were 21.89 mg·g⁻^1^, 1.25 mg·g⁻^1^, 1.20 mg·g⁻^1^, and 3.52 mg·g⁻^1^, respectively, with a pH value of 6.56.

### 4.2. SA Treatments

The randomized block experimental design was set up with four treatments with different concentrations of SA: 0.1 mM, 0.5 mM, 0.9 mM, and 1.3 mM. The water treatment (0 mM SA) was used as the control. The experimental design included three biological replicates for each treatment; a single replicate contained 10 seedlings for a total of 150 plants. During the experimental period, fully automatic sprinkler irrigation was carried out twice a day, and every 15 days, the entire plant’s leaves were sprayed with the corresponding concentration of SA at 10:00 a.m. until the liquid started to drip from the leaf surfaces, and no further water sprinkler irrigation was carried out on that same day. Weeding and pest management were performed once a week. The experimental duration of this study was 6 months; at the end of the experiment, the test materials were collected for analysis of the key components.

### 4.3. Experimental Materials

#### 4.3.1. Growth and Biomass Measurements

The basal diameter (BD, stem diameter at ground level) of all seedlings was measured using a digital vernier caliper with an accuracy of 0.01 mm. Three healthy and uniformly growing seedlings from each treatment were whole-plant excavated and then divided into three parts: leaves, stems, and roots, washed and dried. Each organ of a single plant was dried at 80 °C, and the dry weight of each organ was used as root biomass (RB), stem biomass (SB), leaf biomass (LB), and total plant biomass (TB), expressed as the sum of the dry weight of roots, stems, and leaves. All samples were retained for selection in further analysis.

#### 4.3.2. Endogenous Hormone Measurements

The endogenous hormones indole-3-acetic acid (IAA), abscisic acid (ABA), gibberellin A_3_ (GA_3_), and trans-zeatin-riboside (ZR) were analyzed using an enzyme-linked immunosorbent assay (ELISA) referring to the protocols described by Wu Yu et al. [50]. The ELISA test kits used for the experimental procedure were kindly supplied by the Chongqing Bonoheng Company. The fresh leaves (0.1 g) were rinsed in ice-cold phosphate buffered saline (PBS), dried on filter paper, accurately weighed, and placed in a 5 mL homogenizer tube for manual homogenization. Then, 10% of the homogenate was centrifuged at a speed of 3000 rpm for 10 min. The supernatant was removed for analysis. Three independent biological replicates were established per treatment, each analyzed in triplicate.

#### 4.3.3. Measurements of N, P, and K Content

The dried root, stem, and leaf samples were ground and passed through an 80-mesh sieve to determine nutritional and medicinal components. Referring to Soil Agrochemical Analysis [51], each sample (0.1 g) was weighed and the solution to be tested was obtained after high-temperature digestion. Then, total nitrogen (N) content was determined using continuous flow analysis (AA3, SEAL Analytical, Norderstedt, Germany), total phosphorus (P) content was obtained using molybdenum–antimony colorimetry (INFINITE M200 PRO, Tecan Group Ltd., Männedorf, Switzerland), and total potassium (K) was measured using flame photometry (FP6450, Shanghai Yidian Analytical Instrument, Shanghai, China). Three biological replicates were established for each treatment, again with triplicate analyses.

#### 4.3.4. Analysis of Total Phenolic and Total Flavonoid Content

Total phenolic content in roots (RTPC), stems (STPC), and leaves (LTPC) was determined using the Folin–Ciocalteu method [21,52] in pulverized dried plant tissues: the ethanol extract of each treated sample was thoroughly mixed with forintol reagent (5.0 mL) and 7.5 sodium carbonate solution (4.5 mL), and the optical density (OD) or absorbance value of the solutions were determined at 760 nm. The total phenol content was calculated using Equation (1) and the concentration was determined using the standard curve (y = 6.7079x + 0.0587) established at 760 nm following Beer’s law with gallic acid standard solutions (0.010, 0.020, 0.030, 0.040, and 0.050 mg/mL). Three biological replicates were established for each treatment and also analyzed in triplicate.

(1) $$\mathrm{TPC}\left(\mathrm{mg}\cdot g^{-1}\;\mathrm{DW}\right)=\frac{C\times V\times n}{M}$$

where DW is the dry weight of different organs, C is the concentration in mg/mL determined from a gallic acid standard curve, M stands for the dried weight for extraction in g, and V represents the volume of total extracted solution in mL.

Total flavonoid content in roots (RTFC), stems (STFC), and leaves (LTFC) was determined using dried plant powder samples. The method of Jia Tang was referred to for the determination of TFC [53]: the ethanolic extract of each treated sample was sequentially added to a solution containing 5% sodium nitrite (1 mL), 10% aluminum nitrate (1 mL), and 4% sodium hydroxide (10 mL) to obtain the final solution for measurement. The solution was kept in the dark for 1 h to avoid oxidation of the reaction mixture, and finally, its absorbance was measured at a 510 nm wavelength. Analysis of total flavonoids was carried out using Equation (2) and the standard curve of rutin (y = 5.7628x + 0.0465) established at 510 nm following Beer’s law with standard rutin solutions (0.008, 0.016, 0.032, 0.048, and 0.064 mg/mL). Three biological replicates were established for each treatment, with three triplicates.

(2) $$\mathrm{TFC}\left(\mathrm{mg}\cdot g^{-1}\;\mathrm{DW}\right)=\frac{C\times V\times n}{M}$$

where DW is the dry weight of different organs, C is the concentration in mg/mL determined from the Rutin standard curve, M represents the dried weight for explant extraction in g, and V stands for the volume of total extracted solution in mL.

Equations (3) and (4) were used to calculate total phenolic production (TPP) and total flavonoid production (TFP) of whole plants, respectively.

(3) $$\mathrm{TPP}\left(\mathrm{mg}\;\mathrm{DW}\right)=\mathrm{RTPC}\;\times \;\mathrm{RB}+\mathrm{STPC}\;\times \;\mathrm{SB}+\mathrm{LTPC}\;\times \;\mathrm{LB}$$

(4) $$\mathrm{TFP}\left(\mathrm{mg}\;\mathrm{DW}\right)=\mathrm{RTPC}\;\times \;\mathrm{RB}+\mathrm{STPC}\;\times \;\mathrm{SB}+\mathrm{LTPC}\;\times \;\mathrm{LB}$$

where TPP is the total phenolic production in mg, TFP is the total flavonoid production in mg, DW is the dry weight of the plant, RB is in g, STPC is in mg·g^−1^, SB is in g, LTPC is in mg·g^−1^, and LB is in g.

### 4.4. Data Analysis

The membership function analysis [54] was used to comprehensively evaluate the yield of *M. macrocarpa* under different SA treatments, using the following expressions:

(5) $$\mathrm{Positively}\;\mathrm{correlated}:\;U(X_{i})=\frac{\left(X_{i}-X_{\mathrm{mi}}\right)}{\left(X_{\max}-X_{\min}\right)}$$

(6) $$\mathrm{Negatively}\;\mathrm{correlated}:\;U\left(X_{i}\right)=1-\frac{\left(X_{i}-X_{\min}\right)}{\left(X_{\max}-X_{\min}\right)}$$

where $U(X_{i})$ is the value of the membership function of an indicator, $X_{i}$ stands for the measured value of the index, $X_{min}$ is the minimum value of the index, and $X_{max}\;$represents the maximum value of the index.

The experimental data were collated and statistically evaluated using Excel 2016 software, and one-way analysis of variance and Pearson correlation analysis using IBM SPSS Statistics 27.0 software; graphics and data curves were plotted using Origin 2024 Pro software.

## 5. Conclusions

The study has demonstrated that SA application during *M. macrocarpa* cultivation significantly increased total phenolic and flavonoid contents as well as their yield. This effect was attributed to SA-mediated coordination of hormone signaling and nutrient partitioning across plant organs, which synchronously promoted biomass accumulation and secondary metabolite biosynthesis. These findings expand the theoretical framework of SA’s “hormone–nutrient yield” regulatory role in medicinal plants, and provide practical strategies for improving medicinal plant yields as well as reducing agricultural pollution. Future research should extend SA applications to other medicinal plants, establishing its function as a green elicitor in standardized cultivation. Such innovations could enable large-scale production of high-yield medicinal plants, ultimately benefiting human health through abundant medicinal plant resources.

## Figures and Tables

**Table 1:** Effects of SA concentrations on the growth of *M. macrocarpa*.

SA Concentration (mM)	Basal Diameter (mm)	Biomass (g)			Total Biomass (g)
		Root	Stem	Leaf	
0	6.89 ± 0.49 Bc	16.31 ± 0.90 Cd	19.39 ± 0.73 Bc	17.22 ± 1.00 Bc	52.93 ± 1.66 Dd
0.1	7.09 ± 0.22 ABbc	19.46 ± 0.76 Bc	20.31 ± 0.71 Bc	20.72 ± 0.99 Bb	60.49 ± 1.37 Cc
0.5	7.27 ± 0.40 ABbc	21.85 ± 1.17 Bb	25.10 ± 1.39 Ab	19.83 ± 1.40 Bb	66.79 ± 1.97 Bb
0.9	8.31 ± 0.22 Aa	21.73 ± 1.80 Bb	27.97 ± 1.41 Aa	26.19 ± 1.94 Aa	75.89 ± 0.88 Aa
1.3	7.85 ± 0.74 ABab	25.36 ± 1.06 Aa	27.77 ± 1.30 Aa	25.51 ± 1.44 Aa	78.64 ± 2.13 Aa

Note: Data are expressed as mean ± standard deviation of 3 biological replicates; different capital letters in the same column represent significant differences under different precipitation treatments (*p* < 0.01); different lowercase letters indicate significant differences under different precipitation treatments (*p* < 0.05).

**Table 2:** Effects of different treatments on the endogenous hormone content of *M. macrocarpa* leaves.

SA Concentration (mM)	IAA (ng·g^−1^·FW)	ABA (ng·g^−1^·FW)	GA_3_ (ng·g^−1^·FW)	ZR (ng·g^−1^·FW)
0	46.11 ± 3.72 BCc	156.56 ± 3.97 ABa	4.84 ± 0.14 Cc	5.97 ± 0.10 Bb
0.1	51.47 ± 2.03 Bb	126.16 ± 0.24 Cc	6.10 ± 0.13 Aa	8.98 ± 0.10 Aa
0.5	57.08 ± 0.59 Aa	160.14 ± 3.84 Aa	5.57 ± 0.10 Bb	8.90 ± 0.07 Aa
0.9	42.27 ± 1.25 Cd	154.73 ± 1.38 ABa	5.66 ± 0.19 Bb	9.13 ± 0.41 Aa
1.3	46.47 ± 1.22 BCc	148.96 ± 2.99 Bb	4.90 ± 0.13 Cc	6.22 ± 0.07 Bb

Note: Data are expressed as mean ± standard deviation of three biological replicates, FW is the fresh weight of leaves; different capital letters in the same column represent significant differences under different precipitation treatments (*p* < 0.01); different lowercase letters indicate significant differences under different precipitation treatments (*p* < 0.05).

**Table 3:** Changes in IAA:ABA, ZR:ABA, GA_3_:ABA, and (IAA + GA_3_ + ZR):ABA ratios of *M. macrocarpa*.

SA Concentration (mM)	IAA:ABA	GA_3_:ABA	ZR:ABA	(IAA + GA_3_ + ZR):ABA
0	0.2948 ± 0.0283 Ccd	0.0309 ± 0.0016 Cd	0.0382 ± 0.0011 Ce	0.3639 ± 0.0303 Cc
0.1	0.4080 ± 0.0165 Aa	0.0484 ± 0.0011 Aa	0.0712 ± 0.0006 Aa	0.5275 ± 0.0171 Aa
0.5	0.3566 ± 0.0120 Bb	0.0348 ± 0.0013 Bbc	0.0556 ± 0.0015 Bc	0.4470 ± 0.0146 Bb
0.9	0.2732 ± 0.0070 Cd	0.0366 ± 0.0016 Bb	0.0590 ± 0.0022 Bb	0.3688 ± 0.0084 Cc
1.3	0.3120 ± 0.0025 Cc	0.0329 ± 0.0013 BCcd	0.0418 ± 0.0013 Cd	0.3867 ± 0.0014 Cc

Note: Data are expressed as mean ± standard deviation of three biological replicates; different capital letters in the same column represent significant differences under different precipitation treatments (*p* < 0.01); different lowercase letters indicate significant differences under different precipitation treatments (*p* < 0.05).

**Table 4:** Correlation analysis of growth, endogenous hormone, and nutrient indexes with medicinal constituents in different organs of *M. macrocarpa*.

Index	RTPC	STPC	LTPC	RTFC	STFC	LTFC	TPP	TFP
BD	0.032	0.601 *	0.289	0.578 *	0.659 **	0.354	0.671 **	0.649 **
TB	0.243	0.544 *	0.525 *	0.838 **	0.606 *	0.658 **	0.998 **	0.961 **
ABA	−0.500	−0.083	0.322	0.588 *	0.584 *	−0.127	0.135	0.275
IAA	−0.089	−0.669 **	0.525 *	−0.364	−0.574 *	−0.292	−0.216	−0.340
GA_3_	−0.190	0.286	0.258	−0.426	−0.439	−0.410	0.024	−0.259
ZR	−0.348	0.349	0.485	−0.161	−0.186	−0.464	0.198	−0.080
RN	−0.272	0.206	−0.145	−0.200	−0.044	−0.331	−0.141	−0.211
SN	−0.592 *	0.009	−0.399	−0.482	−0.313	−0.720 **	−0.593 *	−0.620 *
LN	−0.285	0.104	−0.187	−0.360	−0.399	−0.472	−0.231	−0.369
RP	0.484	−0.131	−0.117	0.590 *	0.485	0.784 **	0.329	0.575 *
SP	−0.022	−0.677 **	−0.103	−0.869 **	−0.857 **	−0.514	−0.737 **	−0.833 **
LP	−0.350	−0.553 *	0.742 **	0.193	0.055	−0.254	0.012	0.005
RK	−0.385	0.416	0.422	0.346	0.265	−0.157	0.440	0.320
SK	−0.262	0.514 *	−0.096	−0.206	0.040	−0.334	−0.024	−0.162
LK	−0.466	0.403	0.318	0.691 **	0.652 **	−0.063	0.426	0.471

Note: The values presented in the table are Pearson’s correlation coefficients; ** significant at *p* < 0.01; * significant at *p* < 0.05. BD: basal diameter, TB: total biomass. RN, RP, RK: nitrogen (N), phosphorus (P), and potassium (K) content of roots, respectively. SN, SP, SK: nitrogen (N), phosphorus (P), and potassium (K) content of stems, respectively. LN, LP, LK: nitrogen (N), phosphorus (P), and potassium (K) content of leaves, respectively. RTPC, STPC, LTPC: the total phenolic content of the roots, stems, and leaves, respectively. RTFC, STFC, LTFC: the total flavonoid content of the roots, stems, and leaves, respectively. TPP, TFP: total phenol production and total flavonoid production.

**Table 5:** Membership function values of *M. macrocarpa* seedling under different treatments.

Index	0 mM	0.1 mM	0.5 mM	0.9 mM	1.3 mM
BD	0.000	0.140	0.264	1.000	0.674
TB	0.000	0.294	0.539	0.893	1.000
ABA	0.895	0.000	1.000	0.841	0.671
IAA	0.259	0.621	1.000	0.000	0.284
GA_3_	0.000	1.000	0.582	0.653	0.048
ZR	0.000	0.952	0.927	1.000	0.079
RN	0.267	0.302	0.523	0.000	1.000
SN	0.000	0.370	0.503	0.357	1.000
LN	0.664	1.000	0.721	0.866	0.000
RP	0.553	0.000	0.208	0.236	1.000
SP	0.341	0.000	0.242	1.000	0.847
LP	0.296	0.000	1.000	0.158	0.249
RK	0.000	0.105	0.708	1.000	0.217
SK	0.304	0.836	0.266	1.000	0.000
LK	0.556	0.000	0.726	1.000	0.525
Average value	0.276	0.375	0.614	0.667	0.506
Comprehensive sorting	5	4	2	1	3

Note: BD: basal diameter, TB: total biomass. RN, RP, RK: nitrogen (N), phosphorus (P), and potassium (K) content of roots, respectively. SN, SP, SK: nitrogen (N), phosphorus (P), and potassium (K) content of stems, respectively. LN, LP, LK: nitrogen (N), phosphorus (P), and potassium (K) content of leaves, respectively.

## Data Availability

The original contributions presented in the study are included in the article; further inquiries can be directed to the corresponding author.
